# Rapidly progressive coronary atherosclerosis in a young male: a retrospective advanced CCT phenotype analysis

**DOI:** 10.1007/s10554-024-03216-6

**Published:** 2024-08-13

**Authors:** Daniel Lorenzatti, Annalisa Filtz, Azeem Latib, Joseph DeRose, Damini Dey, Daniel S. Berman, Mario J. Garcia, Leandro Slipczuk

**Affiliations:** 1 Cardiology Division, Montefiore Medical Center, Albert Einstein College of Medicine, 111 E 210st Bronx, Bronx, NY 10467 USA; 2 IRCCS Ospedale Ca’ Granda Maggiore Policlinico, Università degli Studi di Milano, Milan, Italy; 3 Department of Cardiovascular and Thoracic Surgery, Montefiore Medical Center/Albert Einstein College of Medicine, Bronx, NY USA; 4 Department of Imaging, Medicine, and Biomedical Sciences, Cedars-Sinai Medical Center, Los Angeles, CA USA

**Keywords:** Young, Coronary artery disease, CCTA, Plaque, Familiar hypercholesterolemia, FH

## Abstract

We present a real-life case of a very young man with multiple risk factors who progressed rapidly from minimally obstructive non-calcified plaque on computed tomography angiography (CCTA) to severe three-vessel coronary disease presenting with STEMI. It questions the reliability of zero coronary calcium in high-risk subgroups like familial hypercholesterolemia, high Lp(a), and the young. While CCTA can accurately visualize non-calcified plaque, its interpretation requires expertise and clinical judgment should consider both imaging and clinical risk factors for management. Advanced plaque quantification, peri-coronary (PCAT), and epicardial (EAT) adipose tissue could help better-stratified patients but the evidence-based clinical application remains unknown.

A 31-year-old male with past medical history of paroxysmal atrial fibrillation, type II diabetes mellitus (on metformin 500 mg BID), hypertension (on metoprolol succinate 50 mg BID), severe hyperlipidemia (on lovastatin 20 mg), and a family history of premature coronary artery disease (CAD) presented with inferior ST-elevation myocardial infarction (STEMI). The emergency coronary angiogram showed acute occlusion of the proximal to mid-right coronary artery (RCA) along with concomitant obstructive disease in the left anterior descending (LAD) and left circumflex (LCX) coronary arteries **(**Fig. 1**)**. The patient underwent successful primary percutaneous coronary intervention (PCI) of the culprit RCA stenosis with 3 drug-eluting stents (DES) and staged PCI of the Obtuse Marginal (OM) with 2 DES in the same hospital admission. Subsequently, he underwent minimally invasive robotic coronary artery bypass surgery (CABG), using a left internal mammary artery graft to the LAD territory, as part of a complete hybrid revascularization strategy. His metabolic profile on admission was the following: low-density lipoprotein cholesterol (LDL-C) 298 mg/dL; high-density lipoprotein cholesterol (HDL-C) 35 mg/dL; non-HDL-C 323 mg/dL; lipoprotein(a) 204 nmol/L, hemoglobin A1C 8.7%. The genetic panel revealed a single mutation (c313 + 1G > A) on the LDLR gene consistent with heterozygous familial hypercholesterolemia (FH). Upon chart review, it was noticed that the patient underwent computed tomography coronary angiography (CCTA) including coronary artery calcium scoring (CAC) three years before (at 28 years old), in the context of palpitations and atypical chest pain, which was reported as normal at that time (CAD-RADS 0 and zero CAC). The CCTA DICOM images were retrieved and retrospectively analyzed using an advanced plaque quantification solution (Autoplaque v2.5, Cedars-Sinai, Los Angeles, CA). All segments of ≥ 2 mm diameter were manually segmented, and plaque quantification was performed according to expert’s recommendations [1]**(**Fig. 2**)**. Despite the absence of any component of calcified plaque (CP) and no evidence of obstructive disease (maximal diameter stenosis 1–24%), the overall amount of non-calcified plaque in the whole coronary tree was estimated as 3,809 mm3 with a low-attenuation plaque component equal to 941 mm3, corresponding to an overall low attenuation plaque (LAP) burden of 14%, implicating an inherently higher risk for future MI. Using the invasive angiogram **(**Fig. 1**)** as reference, the culprit segment along the mid-RCA was identified and independently analyzed **(**Fig. 3**)**. Even though the maximal diameter stenosis in this segment was only 17% by CCTA, there was a significant burden of LAP and positive remodeling suggesting high-risk plaque features on that segment. In addition, pericoronary adipose tissue (PCAT) analysis was also performed in the proximal 40 mm of the three main coronary vessels as published previously **(**Fig. 4**)** [2]. Interestingly, the highest mean attenuation (a potential marker of inflammation) was found in the culprit RCA (-78.2 HU), followed by the LCX (-82.6 HU) and the LAD (-85.9 HU). Using a deep-learning solution (QFAT; v2.0; Cedars-Sinai Medical Center, Los Angeles, CA, USA) the global amount of epicardial adipose tissue (EAT) was also segmented from the non-contrast CAC scan, and estimated as 93 ml, with a mean density of -79 HU (Fig. 5).

**Fig. 1:**
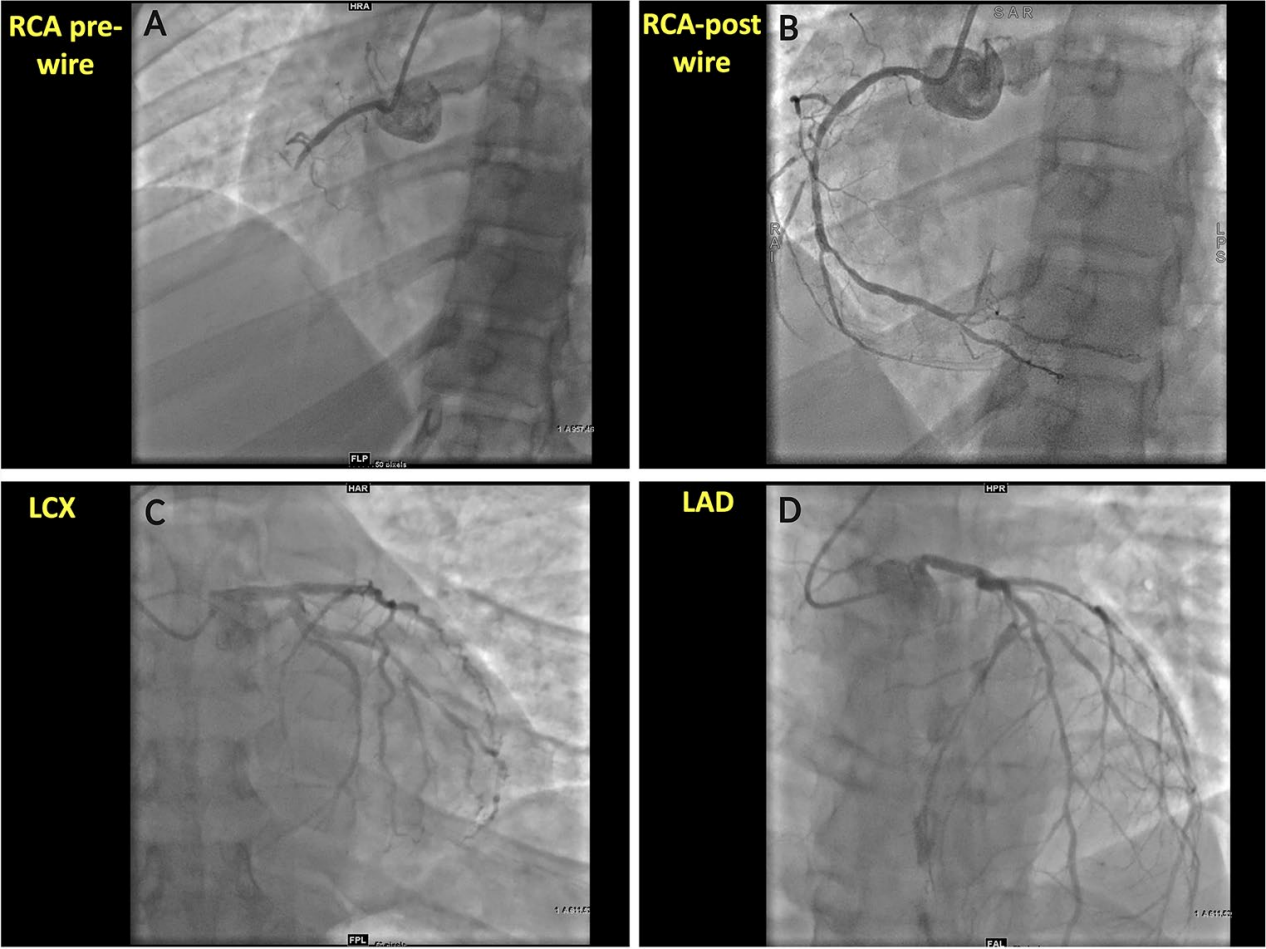
Invasive coronary angiogram at the time of the event. **A**, Dominant right coronary artery (RCA) before wire crossing with acute total occlusion in the mid-segment. **B**, 70% stenosis of mid and mid to distal RCA, 80% stenosis of the distal segment, and 95% stenosis of the posterior-lateral ventricular branch. **C**, Non-obstructive disease of the left circumflex (LCX) artery and 75% stenosis of the 1st Obtuse Marginal (OM1). **D**, 70% stenosis in the mid left anterior descending (LAD) artery and 90% stenosis in the 1st Diagonal (D1)

**Fig. 2:**
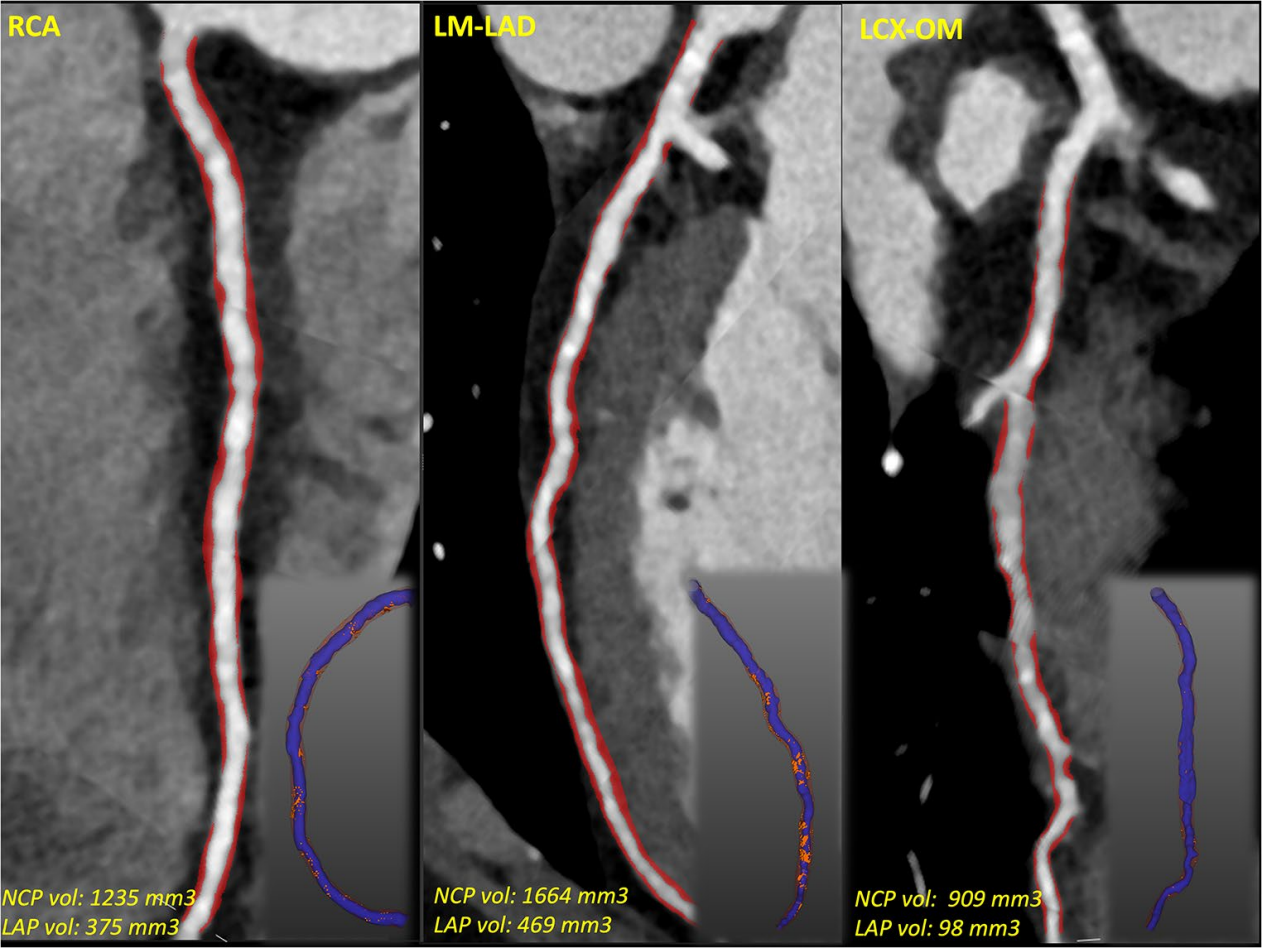
Coronary computed tomography angiography (CCTA) 3 years prior event. Main panels: Curved multiplanar reformations of 3 main epicardial vessels after advanced plaque quantification analysis, showing per-vessel type and volume of plaque. Miniature panels: 3D representation of the corresponding panel vessel depicting lumen in blue, non-calcified plaque (NCP) in red, and low attenuation plaque (LAP) in orange

**Fig. 3:**
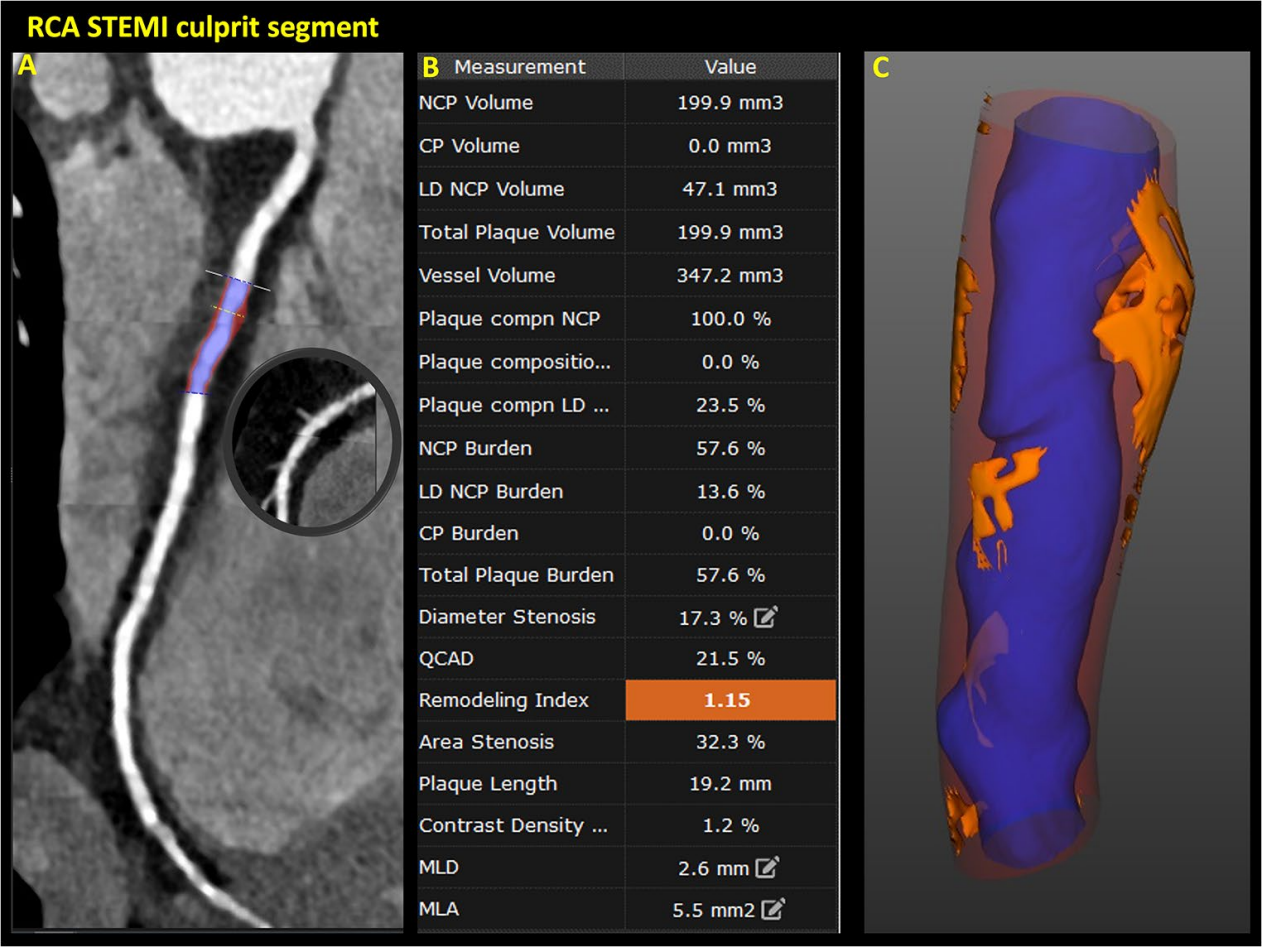
Retrospective CCTA analysis of the presumed RCA culprit segment according to the invasive angiogram. **A**, Curve multiplanar reformat of the RCA showing the analyzed segment. **B**, Table showing all the advanced plaque quantitative features estimated by the software. **C**, 3D representation of the segment showing the positive remodeling and low-attenuation plaque in orange

**Fig. 4:**
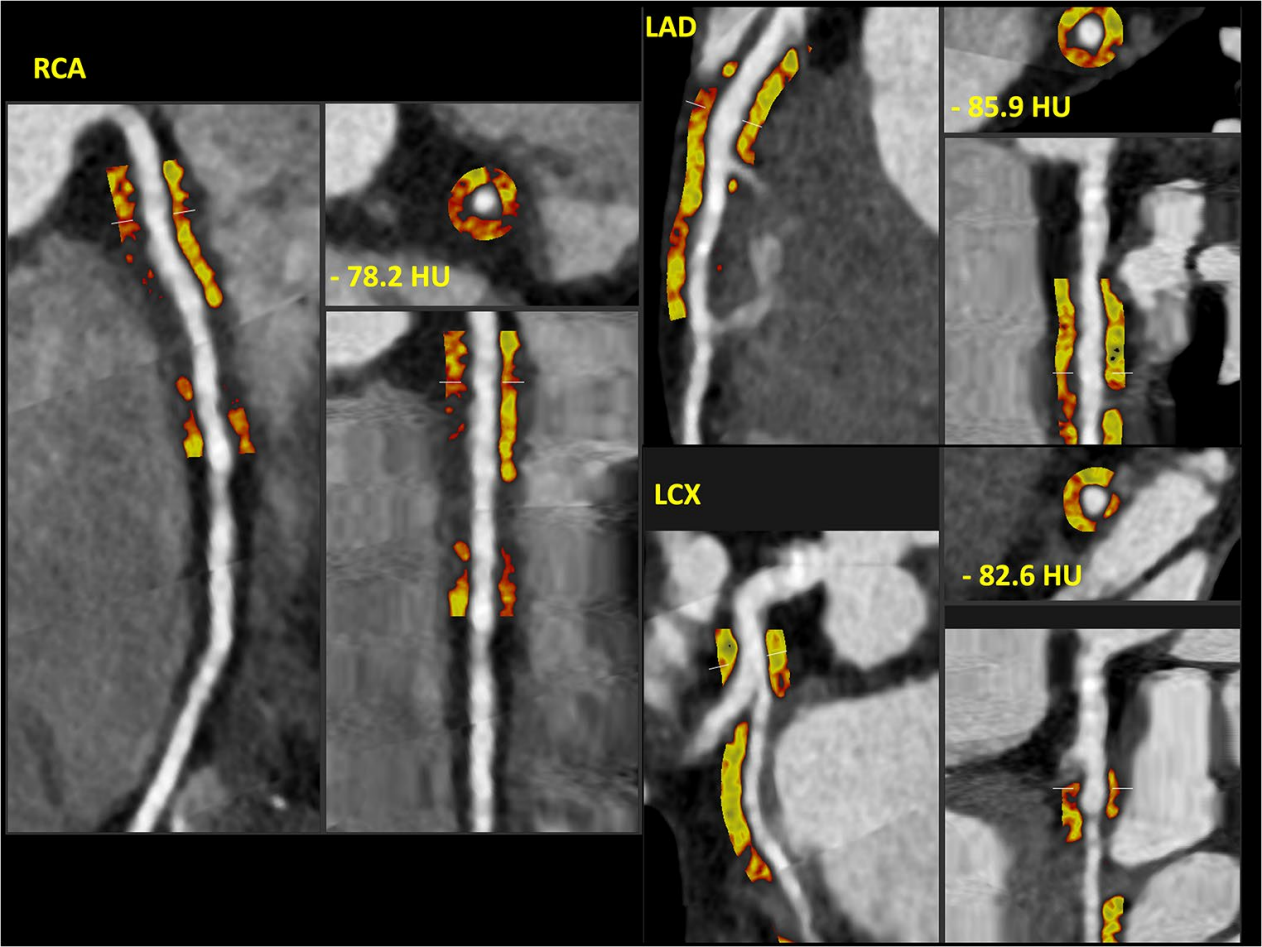
Pericoronary Adipose Tissue (PCAT) analysis involving the proximal segment (40 mm) of the 3 main epicardial coronaries. Average attenuation of the fat tissue within 3 mm of the vessel in Hounsfield Units (HU)

**Fig. 5:**
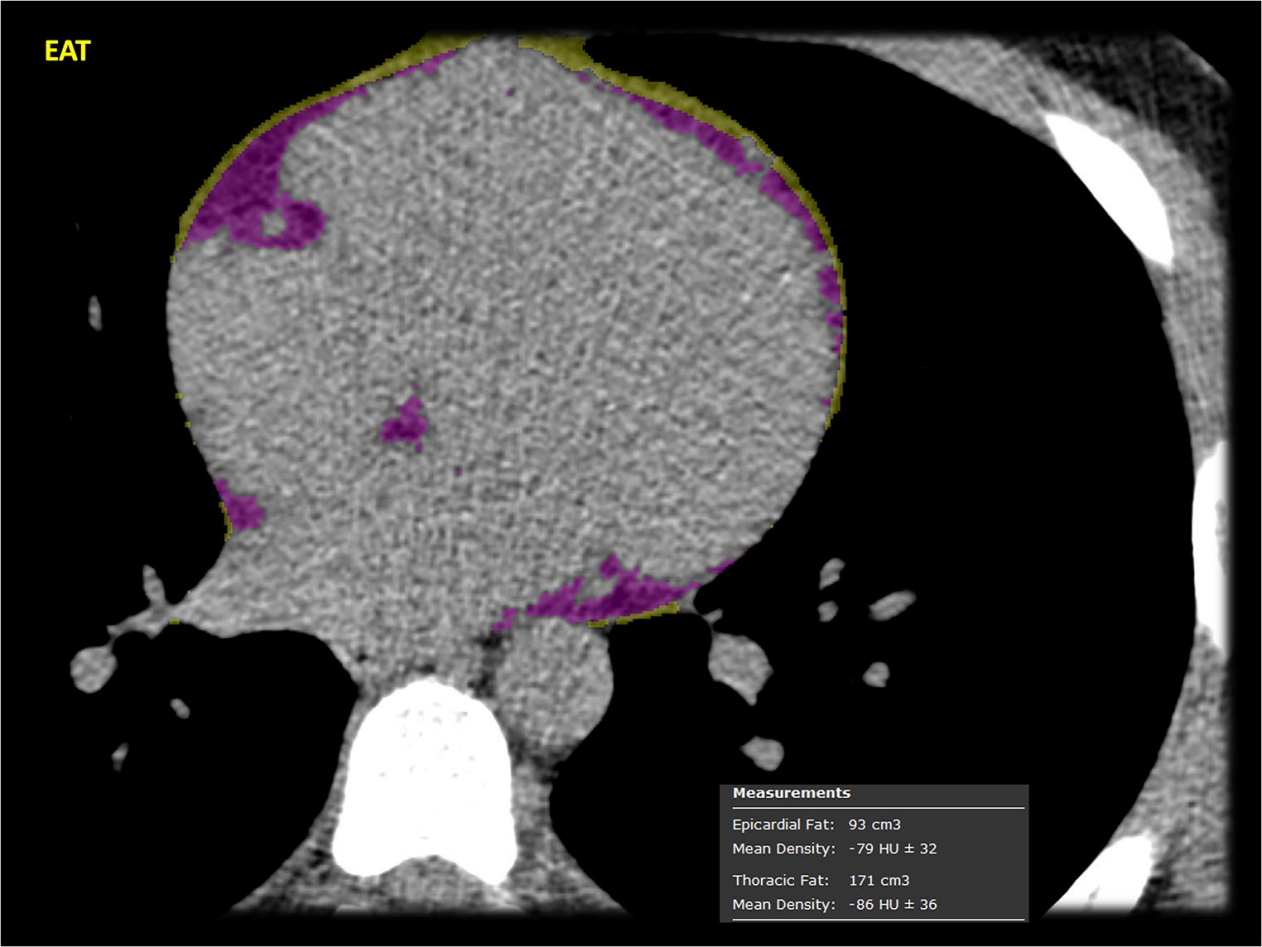
Epicardial Adipose Tissue (EAT) deep-learning quantification and mean attenuation

We present here the case of a young man with several uncontrolled risk factors that despite having a zero CAC and a CCTA with minimally obstructive (1–24% stenosis) non-calcified plaque at baseline, progressed in the lapse of 3 years to multi-vessel obstructive coronary disease, presenting with a STEMI. This case illustrates the limitations of the use of CAC score and the so-called “Power of Zero” for downgrading risk in young and high-risk populations (e.g. FH). We know from CAC population studies that up to 25% of younger patients who suffer a major adverse cardiac event had CAC = 0 at the time of imaging [3]. The main strength of CCTA, besides accurate stenosis evaluation, is the identification of non-calcified plaque and characterization of high-risk plaque morphology, but a sufficient level of expertise is needed, and non-calcified plaque can be overlooked or underestimated (as is this case) without the aid of dedicated vessel reconstructions or advanced plaque quantification solutions. The presence of concomitant cardiovascular risk factors or a family history of premature CAD, should raise the pre-test probability of finding non-calcified plaque and alert the reading physician to perform perhaps a more detailed evaluation, even in young individuals (< 45 years). In addition, it is important to emphasize that risk stratification should always incorporate clinical variables and guideline-directed interventions or pharmacotherapy intensification should not be withheld in patients with uncontrolled risk factors. The proper identification and estimation of the amount of non-calcified and low-attenuation plaque that was already present in the CCTA of this patient 3 years earlier, could have impacted the management in terms of preventive pharmacotherapy intensification and aggressive lifestyle changes. As demonstrated in the SCOT-HEART cohort, the burden of LAP in CCTA is the strongest independent predictor of future fatal or nonfatal myocardial infarction, with a cut-off of LAP > 4% associated with 5-fold risk [4]. The presence of even a minimal amount of plaque at a young age has been associated with an exponential increase in the risk of events [5]. The high volume of plaque plus the burden of the novel risk markers EAT [6] and PCAT [7] could characterize a rapidly progressing atherosclerosis phenotype as seen in this patient. The CCTA semiquantitative plaque burden can be visually estimated as the sum of all diseased coronary segments (Segment Involvement Score, SIS), but despite the proven prognostic value it remains uncommonly reported in clinical practice. An important limitation of SIS is that considers all segments the same no matter the amount of plaque or percentage stenosis, contrary to the advanced plaque quantification solutions [8, 9]. Unfortunately, the integration of these tools into clinical practice remains also limited. Nevertheless, as evidence steadily accrues, together with software iterations that make the process notably expedited and reproducible [10], a near future with widespread clinical application seems increasingly viable. Moreover, data on age, race/ethnicity, and sex-based nomograms for quantitative coronary plaque subtypes as well as CT imaging risk markers is scarce [11], with even less data available in young individuals. It remains to be adequately tested if a deep-learning based approach could significantly impact patient care by better stratifying individuals with diffuse coronary plaque and reducing the possibility of human reader errors.

## Data Availability

No datasets were generated or analysed during the current study.
